# Molecular Diversity of *Giardia duodenalis*, *Cryptosporidium* spp. and *Blastocystis* sp. in Asymptomatic School Children in Leganés, Madrid (Spain)

**DOI:** 10.3390/microorganisms8040466

**Published:** 2020-03-25

**Authors:** Aly Salimo Muadica, Pamela Carolina Köster, Alejandro Dashti, Begoña Bailo, Marta Hernández-de-Mingo, Lucia Reh, Sooria Balasegaram, Neville Q Verlander, Esther Ruiz Chércoles, David Carmena

**Affiliations:** 1 Parasitology Reference and Research Laboratory, National Centre for Microbiology, Health Institute Carlos III, Ctra. Majadahonda-Pozuelo Km 2, 28220 Majadahonda, Madrid, Spain; muadica@gmail.com (A.S.M.); pamelakster@yahoo.com (P.C.K.); dashti.alejandro@gmail.com (A.D.); BEGOBB@isciii.es (B.B.); martaher1@gmail.com (M.H.-d.-M.); LuciaReh@gmx.de (L.R.); 2 European Program for Public Health Microbiology Training, European Centre for Disease Prevention and Control, Gustav III:s Boulevard 40, 169 73 Solna, Sweden; 3 Field Epidemiology Services, National Infection Service, Public Health England, Skipton House, 80 London Road, London SE1 6LH, UK; Sooria.Balasegaram@phe.gov.uk; 4 d Statistics, Modelling and Economics Department, National Infection Service, Public Health England, 61 Colindale Avenue, London NW9 5EQ, UK; Neville.Verlander@phe.gov.uk; 5 Health Care Centre María Jesús Hereza, Jesus Miguel Haddad Blanco 2, 28911 Leganés, Madrid, Spain; eruizc@salud.madrid.org

**Keywords:** *Giardia*, *Cryptosporidium*, *Blastocystis*, *Enterocytozoon*, asymptomatic children, PCR, molecular detection, molecular epidemiology, genotyping, Spain

## Abstract

Enteric parasites including *Giardia duodenalis*, *Cryptosporidium* spp., and to a lesser extent, *Blastocystis* sp. and *Enterocytozoon bieneusi*, are major worldwide contributors to diarrhoeal disease. Assessing their molecular frequency and diversity is important to ascertain the sources of infection, transmission dynamics, and zoonotic potential. Little molecular information is available on the genotypes of these pathogens circulating in apparently healthy children. Here, we show that asymptomatic carriage of *G. duodenalis* (17.4%, 95% CI: 15.5–19.4%), *Blastocystis* sp. (13.0%, 95% CI: 11.4–14.8%), and *Cryptosporidium* spp. (0.9%, 95% CI: 0.5–1.5%) is common in children (1–16 years; *n* = 1512) from Madrid, Spain. Our genotyping data indicate that; (i) the observed frequency and diversity of parasite genetic variants are very similar to those previously identified in Spanish clinical samples, so that the genotype alone does not predict the clinical outcome of the infection, (ii) anthroponotic transmission accounts for a large proportion of the detected cases, highlighting that good personal hygiene practices are important to minimizing the risk of infection, (iii) *Blastocystis* ST4 may represent a subtype of the parasite with higher pathogenic potential, and (iv) *Enterocytozoon bieneusi* does not represent a public health concern in healthy children.

## 1. Introduction

The enteric parasites *Giardia duodenalis* (Metamonada), *Cryptosporidium* spp. (Apicomplexa), *Blastocystis* sp. (Stramenopiles), and *Enterocytozoon bieneusi* (Microsporidia) are among the most frequent diarrheal pathogen affecting humans [1,2]. Transmission of these parasites occurs through the faecal-oral route, either directly from person-to-person or animal-to-person contact, or indirectly through contaminated water or food. Indeed, giardiosis and cryptosporidiosis are predominantly waterborne and foodborne, with outbreaks of both diseases commonly reported globally [3,4]. Not surprisingly, children living in unfavourable settings with poor sanitation and unsafe drinking water are particularly vulnerable to these infections.

Individuals infected by *G. duodenalis*, *Cryptosporidium* spp., *Blastocystis* sp., and *E. bieneusi* may develop a wide range of clinical manifestations ranging from asymptomatic, to acute or chronic diarrheal disease. Clinical signs include diarrhoea, nausea, and weight loss. Vomiting, blood in the stool, and fever are less common [2,5]. There is strong evidence demonstrating that childhood giardiosis and cryptosporidiosis have a strong link with growth and cognitive retardation and failure to thrive [6], even if infected individuals are asymptomatic [7]. Although these conditions have been more frequently reported in children in low-income countries, they have also been identified in industrialized countries, including Spain [8,9]. Additionally, *Blastocystis* sp. infections have been associated with intestinal (irritable bowel syndrome) and extra-intestinal (urticaria) disorders [10,11].

Although rarely a life-threatening condition, about 200 million people have symptomatic giardiasis only in low- and middle-income countries [12]. In contrast, acute *Cryptosporidium* infections are estimated to cause 48,000 annual deaths in children under five years old globally [13]. Regarding *Blastocystis*, more than 1 billion people carry the parasite worldwide [14], whereas *E. bieneusi* infection is regarded as an emerging public health concern both, in immunocompromised (e.g., HIV-infected patients and organ transplant recipients) patients, and immunocompetent individuals [15].

*Giardia duodenalis*, *Cryptosporidium* spp., *Blastocystis* sp., and *E. bieneusi* exhibit extensive intra-species genetic diversity, allowing several genotypes/subtypes to be identified that have marked differences in growth rate, drug susceptibility, host range, geographical distribution, and other biological features. *Giardia duodenalis* is indeed a multi-species complex comprising eight (A to H) distinct assemblages, of which zoonotic assemblages A and B are commonly reported to infect humans [16]. *Cryptosporidium* encompasses at least 38 valid species, with *C. hominis* and *C. parvum* causing more than 90% of documented human cases of cryptosporidiosis [17]. At least 26 subtypes (ST) have been identified within *Blastocystis* sp. Among them, ST1–9 and ST12 have been reported in humans [18]. Finally, several hundred *E. bieneusi* genotypes have been defined and grouped in 11 phylogenetic groups. Group 1 and Group 2 include most of the potentially zoonotic genotypes, whereas the rest of the clusters display genotypes with strong host specificity [2].

It is now clear that most human infections from enteric parasites do not develop any symptoms [19,20]. This issue raises the question of whether pathogenicity and symptomatology may be associated, at least partially, with the parasite’s genotype causing the infection [21,22,23]. In Spain, asymptomatic carriage of diarrhoea-causing enteric parasites has been mainly investigated in schoolchildren populations by conventional (microscopy examination, ELISA) methods [24,25,26]. Molecular-based surveys are far scarcer and restricted to relatively small sample sizes [27]. No information is currently available on the presence of *E. bieneusi* in non-clinical individuals. These studies revealed that asymptomatic carriage of *G. duodenalis*, *Cryptosporidium* spp., and *Blastocystis* sp. have been reported at largely varying (3 to 35%) rates of infection, depending on the population and geographical area surveyed and the diagnostic method used. This PCR and sequencing-based study aims at investigating the occurrence and molecular diversity of *G. duodenalis*, *Cryptosporidium* spp., *Blastocystis* sp., and *E. bieneusi* in a large population of apparently healthy schoolchildren in central Spain.

## 2. Materials and Methods

### 2.1. Ethics Approval and Consent to Participate

This study has been approved by Ethics Committee of the Health Institute Carlos III on 23 October 2017 under the reference number CEI PI17_2017-v3. Informative meetings were held for interested families to explain the purpose of the study and the procedures involved. Written informed consent was obtained from parents or legal guardians of participating schoolchildren.

### 2.2. Study Area and Stool Sample Collection

**Figure 1:**
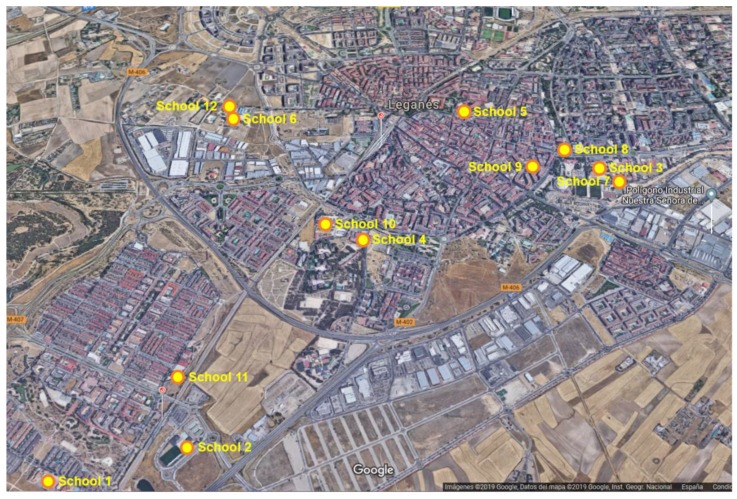
Aerial view of the Leganés municipality (southern metropolitan area of Madrid) indicating the exact geographical location of the 12 primary and secondary schools sampled in the present study.

A cross-sectional molecular epidemiological study of diarrhoea-causing enteric parasites including the protozoan *Giardia duodenalis* and *Cryptosporidium* spp., the stramenopile *Blastocystis* sp., and the microsporidia *Enterocytozoon bieneusi* was carried out in voluntary asymptomatic schoolchildren (3–16 years) in the Leganés municipality (southern metropolitan area of Madrid, central Spain) between November 2017 and June 2018. Stool samples were collected from participating schoolchildren attending 12 primary and secondary schools and their siblings (Figure 1). School features and sampling procedures were described in detail elsewhere [28]. Collected stool samples were transported to the Spanish National Centre for Microbiology and stored at 4 °C (1–5 days) or −20 °C (> 5 days) without preservatives until further PCR testing.

### 2.3. DNA Extraction and Purification

Genomic DNA was isolated from about 200 mg of each faecal specimen by using the QIAamp DNA Stool Mini Kit (Qiagen, Hilden, Germany) according to the manufacturer’s instructions, except that samples mixed with InhibitEX buffer were incubated for 10 min at 95 °C. Extracted and purified DNA samples (200 μL) were kept at −20 °C until further molecular analysis. A water extraction control was included in each sample batch processed.

### 2.4. Molecular Detection and Characterization of Giardia duodenalis

A qPCR protocol amplifying a ~62 bp-fragment of the small subunit ribosomal RNA (*ssu* rRNA) gene of *Giardia duodenalis* was used as initial screening test for the presence of the parasite [29]. The multicopy *ssu* rRNA gene is one of the preferred loci for detection purposes due to its high diagnostic sensitivity. Amplification reactions (25 μL) consisted of 3 μL of template DNA, 0.5 μM of primers Gd-80F and Gd-127R, 0.4 μM of probe (Table S1), and 12.5 μL TaqMan^®^ Gene Expression Master Mix (Applied Biosystems, CA, USA). Detection of parasitic DNA was performed on a Corbett Rotor Gene™ 6000 real-time PCR system (Qiagen) using an amplification protocol consisting on an initial hold step of 2 min at 55 °C and 15 min at 95 °C followed by 45 cycles of 15 s at 95 °C and 1 min at 60 °C. Water (no-template) and genomic DNA (positive) controls were included in each PCR run.

*Giardia duodenalis* isolates with a qPCR-positive result were re-assessed by sequence-based multi-locus genotyping of the single-copy genes encoding for the glutamate dehydrogenase (*gdh*), ß-giardin (*bg*) and triose phosphate isomerase (*tpi*) proteins of the parasite. A semi-nested PCR was used to amplify a ~432-bp fragment of the *gdh* gene [30]. PCR reaction mixtures (25 μL) included 5 μL of template DNA and 0.5 μM of the primer pairs GDHeF/GDHiR in the primary reaction and GDHiF/GDHiR in the secondary reaction (Table S1). Both amplification protocols consisted of an initial denaturation step at 95 °C for 3 min, followed by 35 cycles of 95 °C for 30 s, 55 °C for 30 s and 72 °C for 1 min, with a final extension of 72 °C for 7 min. A nested PCR was used to amplify a ~511 bp-fragment of the *bg* gene [31]. PCR reaction mixtures (25 μL) consisted of 3 μL of template DNA and 0.4 μM of the primers sets G7_F/G759_R in the primary reaction and G99_F/G609_R in the secondary reaction (Table S1). The primary PCR reaction was carried out with the following amplification conditions: One step of 95 °C for 7 min, followed by 35 cycles of 95 °C for 30 s, 65 °C for 30 s, and 72 °C for 1 min with a final extension of 72 °C for 7 min. The conditions for the secondary PCR were identical to the primary PCR, except that the annealing temperature was 55 °C. Finally, a nested PCR was used to amplify a ~530 bp-fragment of the *tpi* gene [32]. PCR reaction mixtures (50 μL) included 2–2.5 μL of template DNA and 0.2 μM of the primer pairs AL3543/AL3546 in the primary reaction and AL3544/AL3545 in the secondary reaction (Table S1). Both amplification protocols consisted of an initial denaturation step at 94 °C for 5 min, followed by 35 cycles of 94 °C for 45 s, 50 °C for 45 s and 72 °C for 1 min, with a final extension of 72 °C for 10 min.

### 2.5. Molecular Detection and Characterization of Cryptosporidium spp. Isolates

The presence of *Cryptosporidium* spp. was assessed using a nested PCR to amplify a ~587-bp fragment of the *ssu* rRNA gene of the parasite [33]. Amplification reactions (50 μL) included 3 μL of DNA sample and 0.3 μM of the primer pairs CR-P1/CR-P2 in the primary reaction and CR-P3/CPB-DIAGR in the secondary reaction (Table S1). Both PCR reactions were carried out as follows: One step of 94 °C for 3 min, followed by 35 cycles of 94 °C for 40 s, 50 °C for 40 s and 72 °C for 1 min, concluding with a final extension of 72 °C for 10 min. Sub-typing of the isolates identified as *C. hominis* or *C. parvum* was attempted at the 60 kDa glycoprotein (*gp60*) gene. Briefly, a nested PCR was conducted to amplify a ~870-bp fragment of the *gp60* locus [34]. PCR reaction mixtures (50 μL) included 2–3 μL of template DNA and 0.3 μM of the primer pairs AL-3531/AL-3535 in the primary reaction and AL-3532/AL-3534 in the secondary reaction (Table S1). The primary PCR reaction consisted of an initial denaturation step of 94 °C for 5 min, followed by 35 cycles of 94 °C for 45 s, 59 °C for 45 s, and 72 °C for 1 min with a final extension of 72 °C for 10 min. The conditions for the secondary PCR were identical to the primary PCR except that the annealing temperature was 50 °C.

### 2.6. Molecular Detection and Characterization of Blastocystis sp. Isolates

Identification of *Blastocystis* sp. was achieved by a direct PCR targeting the *ssu* rRNA gene of the parasite [35]. This protocol uses the pan-*Blastocystis*, barcode primers RD5 and BhRDr (Table S1) to amplify a PCR product of ~600 bp. Amplification reactions (25 μL) included 5 μL of template DNA and 0.5 μM of the primer set RD5/BhRDr. Amplification conditions consisted of one step of 95 °C for 3 min, followed by 30 cycles of 1 min each at 94, 59 and 72 °C, with an additional 2 min final extension at 72 °C.

### 2.7. Molecular Detection and Characterization of Enterocytozoon bieneusi Isolates

*Enterocytozoon bieneusi* DNA was detected using a nested PCR to amplify a ~390 bp-fragment of the entire internal transcribed spacer as well as portions of the flanking large and small subunits of the rRNA gene of the parasite [36]. Reaction mixtures (50 μL) contained one μL of DNA template and 0.2 μM of the primer pairs EBITS3/EBITS4 in the primary reaction and EBITS1/EBITS2.4 in the secondary reaction (Table S1). After denaturation at 94 °C for 3 min, amplification conditions of the primary PCR included 35 cycles of amplification (denaturation at 94 °C for 30 s, annealing at 57 °C for 30 s, and elongation at 72 °C for 40 s), followed by a final extension at 72 °C for 10 min. Conditions for the secondary PCR were identical to the primary PCR except only 30 cycles were carried out with an annealing temperature of 55 °C.

All samples were tested singly. Diagnostic sensitivities of the PCR-based methods used for the detection of *G. duodenalis*, *Cryptosporidium* spp., *Blastocystis* sp., and *E. bieneusi* were over 95%. Diagnostic specificities were near 100% as most of the PCR-positive samples were confirmed by sequencing. All the direct, semi-nested, and nested PCR protocols described above were conducted on a 2720 thermal cycler (Applied Biosystems). Reaction mixes always included 2.5 units of MyTAQ™ DNA polymerase (Bioline GmbH, Luckenwalde, Germany), and 5× MyTAQ™ Reaction Buffer containing 5 mM dNTPs and 15 mM MgCl_2_. Laboratory-confirmed positive and negative DNA isolates for each parasitic species investigated were routinely used as controls and included in each round of PCR. PCR amplicons were visualized on 2% D5 agarose gels (Conda, Madrid, Spain) stained with Pronasafe nucleic acid staining solution (Conda). Positive-PCR products were directly sequenced in both directions using the internal primer set described above. DNA sequencing was conducted by capillary electrophoresis using the BigDye^®^ Terminator chemistry (Applied Biosystems) on an ABI PRISM 3130 automated DNA sequencer.

### 2.8. Sequence and Phylogenetic Analyses

Raw sequencing data in both forward and reverse directions were viewed using the Chromas Lite version 2.1 (Technelysium Pty Ltd., South Brisbane, Australia) sequence analysis program (https://technelysium.com.au/wp/chromas/). The BLAST tool (http://blast.ncbi.nlm.nih.gov/Blast.cgi) was used to compare nucleotide sequences with sequences retrieved from the NCBI GenBank database. Generated DNA consensus sequences were aligned to appropriate reference sequences using the MEGA version 6 software [37] to identify *Giardia* species and assemblages/sub-assemblages and *Cryptosporidium* species. *Blastocystis* sequences were submitted at the *Blastocystis* 18S database (http://pubmlst.org/blastocystis/) for sub-type confirmation and allele identification. The sequences obtained in this study have been deposited in GenBank under accession numbers MN844134–MN844151 (*G. duodenalis*), MN836820–MN836825 (*Cryptosporidium* spp.) and MN836826–MN836842 (*Blastocystis* sp.).

## 3. Results

### 3.1. Occurrence of Enteric Parasites

During the period of study, 1512 stool samples were collected from schoolchildren (age group: 4–14 years, *n* = 1359) attending one of 12 schools in Leganés (Madrid, Spain) and their siblings (age group: 1–16 years, *n* = 153). Only 10 participating children of the latter group had gastrointestinal symptoms the day of sampling and the previous week, but this was not found to be associated with any parasite, so children were still included in the study. Initial prevalence data and assessment of potential risk and/or protective factors associated with parasite infection were thoroughly described elsewhere [28]. Overall, *G. duodenalis* was the most prevalent enteric parasite found (17.4%, 95% confidence interval (CI): 15.5–19.4%), followed by *Blastocystis* sp. (13.0%, 95% CI: 11.4–14.8%), and *Cryptosporidium* spp. (0.9%, 95% CI: 0.5%–1.5%). *Enterocytozoon bieneusi* was not detected in any of the samples analysed. The prevalence rates of these pathogens in each participating school are summarized in Table 1. Estimates did not consider the clustered nature of the data, as this task was thoroughly conducted elsewhere [28]. The full dataset used to determine the prevalence rates and molecular diversity of *G. duodenalis*, *Cryptosporidium* spp., *Blastocystis* sp., and *E. bieneusi* of in this study is presented as a spreadsheet (Table S2).

### 3.2. Molecular Characterization of G. duodenalis Isolates

A total of 263 DNA isolates tested positive for *G. duodenalis* by qPCR. Generated cycle threshold (Ct) values ranged from 18.7–41.1 (median: 33.9; 25th centile: 31.2; 75th centile: 35.3). Of these, 81% (213/263) had qPCR Ct values > 30. Based on previous molecular studies conducted by our research team in Spanish clinical populations [9,38], only DNA isolates with qPCR Ct values ≤ 30 (*n* = 50) were used for genotyping and sub-genotyping purposes.

Out of the 50 DNA isolates investigated, 42% (21/50), 18% (9/50), and 24% (12/50) were successfully amplified at the *gdh*, *bg*, and *tpi* loci, respectively. A total of 24 isolates were genotyped and/or sub-genotyped by any of the three markers. Multi-locus genotyping data were available for 12% (6/50) of the isolates characterised (Table 2). Sequence analyses revealed the presence of assemblages A (17%, 4/24) and B (83%, 20/24). All A sequences were assigned to the sub-assemblage AII of the parasite. Out of the 20 B sequences 19 were assigned to the sub-assemblage BIV and one corresponded to an ambiguous BIII/BIV result. No infections caused by mixed A+B or canine (C, D), feline (F), or ruminant (E) assemblages were detected.

The diversity, frequency and main features of the *G. duodenalis* sequences generated at the *gdh*, *bg*, and *tpi* loci were summarized in Table 3. At the *gdh* locus, three out of four AII sequences were identical to reference sequence L40510, with the remaining one differing from it by a single nucleotide polymorphism (SNP) at positions 269 (a double peak C/T). A much higher level of genetic diversity was observed within the 16 sequences unequivocally identified as sub-assemblage BIV, which differed by none to four SNPs with reference sequence L40508. Out of these BIV sequences, six were identical to L40508, eight contained ambiguous C/T sites (including heterozygous positions in the form of double peaks) at positions 183, 387, 396, and/or 423 of L40508, and two contained SNPs other than the above-mentioned. An additional sequence corresponded to a discordant BIII/BIV typing result (probably reflecting a BIII+BIV intra-assemblage mixed infection) differing by 12 SNPs (all of them corresponding to double peaks in the electropherogram) with L40508 (Table 3).

Similar results were observed with the sequences generated at the *bg* locus. All three AII sequences showed 100% identity with the reference sequence AY072723, whereas the six sequences ascribed to the assemblage B varied from six to seven SNPs with reference sequence AY072727. Most of these B sequences included a typical pattern of mutations at positions 159 (G/A), 165 (C/T), 309 (C/T), 324 (C/T), 393 (C/T), and 471 (T/C) with minor variations (Table 3). At the *tpi* locus two out of the three AII sequences identified were identical to reference sequence U57897, with the remaining one differing from it at positions 287 (C/G) and 291 (A/W). Seven out of the nine BIV sequences showed 100% identity with reference sequence AF069560. The remaining two sequences varied from it by one to three SNPs. BIV sequences generated at the *tpi* locus had comparatively lower genetic diversity than their counterparts did at the *gdh* and *bg* loci (Table 3).

### 3.3. Molecular Characterization of Cryptosporidium spp. Isolates

Analyses of the 14 *ssu* rDNA sequences generated in the present study revealed the presence of two *Cryptosporidium* species circulating in the schoolchildren population investigated, including *C. hominis* (71%; 10/14) and *C. parvum* (21%; 3/14). An additional sample (1/14) was only identified at the genus level due to insufficient sequence quality (Table 4). A remarkable degree of genetic variability was observed among the 10 sequences assigned to *C. hominis*. Only five of them were identical to reference sequence AF108865, whereas the remaining five differed from it by one to four SNPs including substitutions, insertions, and the deletion of multiple nucleotides (Table 4). Two of the three sequences assigned to *C. parvum* revealed 98% identity with reference sequence AF112571, although their quality was insufficient to ascertain with accuracy the presence/absence of SNPs. One of them was demonstrated to belong to the *gp60* genotype family IId. The third sequence was identified as the ´bovine genotype´ of *C. parvum*, also known as *C. pestis* by some authors [39] (Table 4).

### 3.4. Molecular Characterization of Blastocystis sp. Isolates

**Figure 2:**
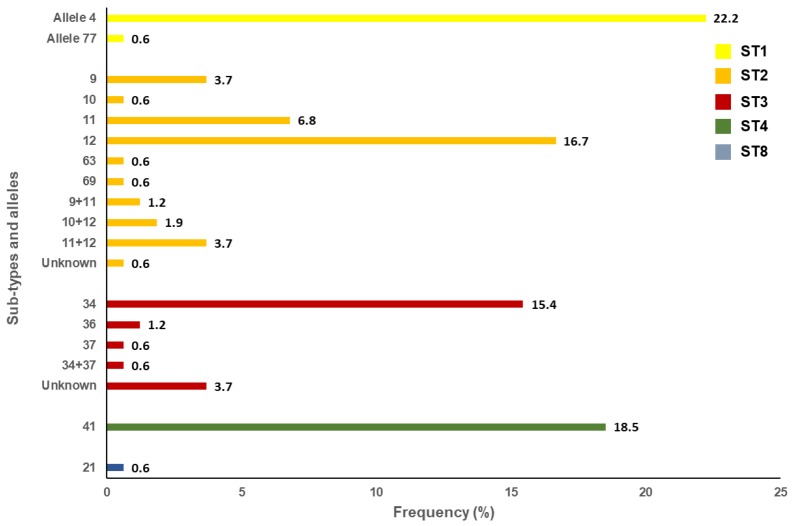
Diversity and frequency of *Blastocystis* subtypes and 18S alleles identified in the schoolchildren population surveyed in the present study, Leganés, Madrid, 2017–2019.

Out of the 197 isolates that tested positive for *Blastocystis* sp. by PCR, 82% (162/197) were successfully subtyped. The remaining 35 isolates produced unreadable or poor-quality sequences typically associated to faint bands on agarose gels. Sequence analyses at the *ssu* rDNA (barcode region) gene of the parasite revealed the presence of five *Blastocystis* subtypes (ST), including ST1 (23%; 37/162), ST2 (36%; 59/162), ST3 (22%; 35/162), ST4 (19%; 30/162), and ST8 (1%; 1/162) (Figure 2). Neither mixed infection involving different STs of the parasite nor infections caused by animal-specific ST10-ST17 were identified. A large genetic diversity was observed within ST2 (alleles 9, 10, 11, 12, 63, 69. 9+11, 10+12, and 11+12) and ST3 (alleles 34, 36, 37, and 34+37). In contrast, only alleles 4 and 77 were observed within ST1, allele 42 within ST4, and allele 21 within ST8. Seven isolates (one in ST2 and six in ST3) could not be analysed at the allele level due to inaccurate or incomplete sequencing data (Figure 2).

No obvious clusters of parasite´s species/genotypes were found according to the period of sampling or the sex, age group, and school of origin of the infected children, investigated in the present study.

## 4. Discussion

In the present study asymptomatic carriage of *G. duodenalis* (17%), *Blastocystis* sp. (13%), and *Cryptosporidium* spp. (0.9%), but not *E. bieneusi*, was reported in apparently healthy schoolchildren in Leganés (Madrid); although unadjusted for clustering within schools, this is likely to be a fair estimate. This is the largest PCR and sequencing-based molecular survey conducted in asymptomatic individuals in Spain to date. Molecular data generated here were strengthened by the adoption of a multi-locus genotyping scheme for the molecular characterization of samples positive to *G. duodenalis* and *Cryptosporidium* spp.

In Spain, asymptomatic carriage of *G. duodenalis* has been previously reported in pre-schoolchildren (prevalence: 15–25%) and schoolchildren (prevalence: 3–36%) populations from rural and urban areas by conventional methods including light and immunofluorescence microscopy, ELISA for the detection of copro-antigens or rapid diagnostic tests [24,27,40]. Genotyping data are, however, far scarcer. Assemblage B (57%) was the predominant *G. duodenalis* genetic variant detected in schoolchildren in the Álava province (northern Spain), followed by sub-assemblage AII (29%) and AII+B mixed infections (14%) [27]. BIV was the only sub-assemblage found in an ensuing survey conducted in the same region [41], and in children attending day care centres in the Madrid province, central Spain [40].

*Giardia duodenalis* prevalence (17%) and genotyping (AII: 17%; BIV: 79%: BIII/BIV: 4%) data presented here agreed well with the figures reported in the studies mentioned above, corroborating that assemblage B is 2–3 fold more prevalent than assemblage A in Spanish asymptomatic children populations. This is in sharp contrast with the results obtained in a recent molecular study conducted in *G. duodenalis*-positive outpatients of all ages attending hospital settings in 10 Spanish provinces showing that asymptomatic infection was more common in individuals with assemblage A than in those with assemblage B (14% *versus* 1.5%, *n* = 29) [42]. Strikingly, the *G. duodenalis* genotype frequencies shown here are very similar to those (AII: 15%; BIV: 62%: BIII/BIV: 1.6%, *n* = 124), previously reported in patients with gastrointestinal complaints attending two major public hospitals in the Madrid province [38]. Not coincidentally, one of these hospitals was the University Hospital Severo Ochoa located in Leganés, whose catchment area was the very same investigated in the present study. It should be highlighted that these similitudes were also present at the nucleotide level, as *gdh* and *bg* sequence variants, generated in both studies, were observed at similar proportions. Overall, these data seem to indicate that essentially the same *G. duodenalis* genotypes circulate in asymptomatic carriers and symptomatic patients in Madrid province. Different assemblage A/B ratios have been identified in other Spanish regions [9,43], suggesting that the frequency and diversity of *G. duodenalis* genotypes may be geographically dependent.

In this study the presence of *Cryptosporidium* spp. was confirmed in a very low proportion (<1%) of asymptomatic schoolchildren. Infection rates ranging from 1% to 10% have been previously estimated by non-molecular methods in children attending day care centres in Madrid [40] and the Salamanca province, western Spain [44], and in schoolchildren in the Álava province [27,41]. When available, molecular data in these surveys showed the presence of *C. hominis* only [27]. Our sequence analyses revealed that *C. hominis* was more prevalent than *C. parvum* (71% *versus* 21%, *n* = 14). Similar proportions have been previously described in clinical samples in different Spanish regions including Barcelona (88% *versus* 10%, *n* = 69) [45], Galicia (65% *versus* 34%, *n* = 486) [46], La Rioja (82% *versus* 19%, *n* = 81) [9], and Madrid (82% *versus* 13%, *n* = 108) [47]. These clinical studies also revealed that Ib (predominantly IbA10G2) and IIa (predominantly IIaA15G2R1) were the most frequent *gp60* genotype families within *C. hominis,* and *C. parvum*, respectively. As in the case of *G. duodenalis*, these data indicate that *C. hominis* and *C. parvum* are circulating in both asymptomatic and symptomatic (clinical) populations in Spain. Absence of canine- (*C. canis*), feline- (*C. felis*), livestock (*C. andersoni*, *C. bovis*) or avian-specific (*C. meleagridis*) *Cryptosporidium* species seem to suggest that the spreading of the infection in the paediatric population under study is mainly through a human transmission cycle. Notably, *C. hominis* has also been more prevalently found than *C. parvum* in low- and medium-income countries with insufficient sanitation facilities [48].

The finding that *G. duodenalis* assemblages/sub-assemblages and *Cryptosporidium* species/genotype families are present at approximately equal proportions in both apparently healthy individuals and in subjects presenting with clinical manifestations in Spain has profound implications. It indicates that genotype does not suffice to explain the outcome of an infection by these pathogens, or the reason the very same pathogen can be virulent in one host, but avirulent in another. Microbial virulence has been considered as an emergent property, implying that the consequence of host-microbe interaction is inherently unpredictable [49]. In this regard, the term pathobiont has been initially coined to describe bacteria that colonize the gastrointestinal tract of individuals asymptomatically, but also have the potential to cause disease [50]. Mounting evidence demonstrates that the gut microbiota play a crucial role in host resistance against invading pathogens and indigenous pathobionts within the intestine through a number of mechanisms, including competitive metabolic interactions, localization to intestinal niches, and induction of host immune responses [51]. We hypothesize that *G. duodenalis* and *Cryptosporidium* spp., and very likely other enteric protists, may act as pathobionts under certain circumstances, and that their interaction with the host´s microbiome and immune system may modulate their pathogenic responses. Definitively more research should be conducted in this interesting area of knowledge.

The *Blastocystis* sp. carriage rate estimated here (13%) was well in the range of those (8%–23%) previously reported by microscopy examination in preschool- and schoolchildren in Salamanca province [52]. Infection rates identified in earlier clinical studies in the country ranged from 3%–7% in symptomatic outpatients to 10% in HIV-infected children [53,54]. In Europe, ST1 to ST4 are the most common *Blastocystis* subtypes circulating in humans [55]. This is also the case of the present study, where ST2 (36%), was the most prevalent genetic variant of the parasite, followed by ST1 (23%), ST3 (22%) and ST4 (19%). The finding of ST8 (0.6%) can be considered as sporadic. These data indicate that the spreading of *Blastocystis* sp. in the paediatric population investigated is primarily through a human transmission cycle. Notably, the same results (although with a higher prevalence rate of 62% for ST2) have been documented in a community survey involving 179 individuals of all ages conducted in Álava province [26]. In both surveys, ST4 appeared to be considerably less represented (7.4–19%) than ST1-ST3. Interestingly, ST4 has been identified in 94% of *Blastocystis* mono-infected patients with diarrhoea in Valencia province, central-east Spain [23]. Similar findings have been reported in patients presenting with acute diarrhoea in Denmark [56] and in patients suffering from irritable bowel syndrome (IBS) and chronic diarrhoea in Italy [57]. Additionally, the relative frequency of ST4 seems to be higher than those for ST1-ST3 in patients with diarrhoea in hospital settings in Spain [58]. Taken together, these data suggest that ST4 could be more pathogenic than other *Blastocystis* STs. In support of this hypothesis, human acquisition of the ST4 lineage, very likely from wild rodents, has been proposed as a relatively recent evolutionary event [59]. Poor host adaptation may result, therefore, in increased parasite virulence. However, all these lines of evidence should still be considered with caution, as other investigations failed to demonstrate the pathogenic nature of ST4 [60,61]. Moreover, other research groups have proposed a link between ST1 and the aetiology of IBS [62], or between ST3 and gastrointestinal disorders, including diarrhoea [63].

It is well known that ST5 to ST9 are reported in humans only rarely [55]. In the present study ST8 was identified in a 10-year-old female reporting no contact with companion animals and no obvious risk factors for parasite infections. ST8 carriage has been previously documented almost exclusively in captive and free-living non-human primates in Central America [64,65], South America [66,67], and Europe [68]. Remarkably, an unexpectedly high prevalence of ST8 was seen among primate handlers in a zoological garden in UK, suggesting that zoonotic transmission of *Blastocystis* ST8 infections from primates to their handlers had occurred [68]. In our study, the source of infection by ST8 remains unclear.

Intriguingly, the microsporidia *E. bieneusi* was apparently absent in the surveyed, apparently healthy, schoolchildren population. In the only published study conducted by PCR in asymptomatic healthy individuals, *E. bieneusi* was detected in 6.0% (23/382) of people of all age groups living in the Czech Republic [69]. In that survey only four out of the 23 detected cases of microsporidiosis by *E. bieneusi* occurred in children younger than 12 years of age. Taken together, these data are indicative of an age-related pattern of infection, with older individuals being more likely to harbour the parasite than younger ones. To date, *E. bieneusi* infections in Spain have been only identified in HIV/AIDS patients [54,70], transplant recipients [71], returning travellers [72], elderly people [73], and immunocompetent clinical populations [74].

This study presents some limitations. For instance, the different PCR formats (qPCR, nested PCR, direct PCR) used very likely differ in their diagnostic performances. In practical terms this means that the reported prevalence rates for certain parasites (e.g., *Blastocystis* sp.) may be underestimated. A relatively low proportion of *G. duodenalis*-positive samples were successfully genotyped at the assemblage and sub-assemblage level. This is a direct consequence of the high Ct values obtained by qPCR in most of the samples tested, a fact that compromised the diagnostic performance of the (single-copy gene) PCR methods used for genotyping purposes. The same is also true for the failure to determine *C. hominis*/*C. parvum* subtypes at the *gp60* marker. These are somehow expected results, as asymptomatic carriage of intestinal parasites correlates well with light infections. Regarding Microsporidia, this study focuses exclusively on *E. bieneusi*. However, previous studies have revealed that microsporidia species, belonging to the *Encephalitozoon* genus, were frequently found in asymptomatic, apparently healthy individuals [69]. Investigating the presence of *Encephalitozoon* spp. should be investigated in future surveys.

## 5. Conclusions

This is the largest PCR-based epidemiological study investigating the genetic variability of *G. duodenalis*, *Cryptosporidium* spp., *Blastocystis* sp., and *E. bieneusi* in apparently healthy schoolchildren conducted in Spain to date. Molecular data presented here reveal important new insights in the epidemiology of these parasites. First, the observed frequency and diversity of species/genotypes are very similar to those previously identified in clinical samples. This finding strongly suggests that the genotypes of these pathogen are not determinant in tilting the balance between health and disease, and that other factors (e.g., co-infections, microbiome composition, and host immune status), very likely play a role in it. Second, anthroponotic transmission accounts for a large proportion of the detected cases, highlighting that improvement of personal hygiene practices (e.g., hand washing) is essential to minimize the risk of infection. Third, *Blastocystis* ST4 may represent a subtype of the parasite with higher pathogenic potential. Fourth, *E. bieneusi* does not represent a public health concern in healthy children.

## Figures and Tables

**Table 1:** Prevalence (%) and 95% confidence intervals (CIs) of the enteric parasite species investigated in the present survey according to school of origin, Leganés, Madrid, 2017–2019.

School	*n*	*Giardia duodenalis*	*Cryptosporidium* spp.	*Blastocystis* sp.	*Enterocytozoon bieneusi*
Global	1512	17.4 (15.5–19.4)	0.9 (0.5–1.5)	13.0 (11.4–14.8)	0.0 (NA)
1	233	25.8 (20.3–31.9)	0.0 (0.0–0.0)	17.6 (12.9–23.1)	0.0 (NA)
2	134	26.1 (18.9–34.4)	0.0 (0.0–0.0)	11.9 (7.0–18.7)	0.0 (NA)
3	124	20.2 (13.5–28.3)	0.8 (0.0–4.4)	9.7 (5.1–16.3)	0.0 (NA)
4	54	7.4 (2.1–17.9)	1.9 (0.0–9.9)	3.7 (0.5–12.7)	0.0 (NA)
5	65	1.5 (0.0–8.3)	0.0 (0.0–0.0)	13.8 (6.5–24.7)	0.0 (NA)
6	144	9.0 (4.9–14.9)	0.0 (0.0–0.0)	13.2 (8.1–19.8)	0.0 (NA)
7	214	11.2 (7.3–16.2)	3.7 (1.6–7.2)	14.5 (10.1–19.9)	0.0 (NA)
8	73	11.0 (4.9–20.5)	1.4 (0.0–7.4)	15.1 (7.8–25.4)	0.0 (NA)
9	142	9.9 (5.5–16.0)	0.0 (0.0–0.0)	12.0 (7.1–18.5)	0.0 (NA)
10	136	30.9 (23.2–39.4)	1.5 (0.2–5.2)	11.8 (6.9–18.4)	0.0 (NA
11	128	19.5 (13.1–27.5)	0.0 (0.0–0.0)	10.2 (5.5–16.7)	0.0 (NA)
12	65	18.5 (9.9–30.0)	1.5 (0.0–8.3)	15.4 (7.6–26.5)	0.0 (NA)

NA: not applicable.

**Table 2:** Genotyping results of *Giardia duodenalis* sequences at the *gdh*, *bg* and *tpi* loci obtained in the children population under study in Leganés, Madrid, 2017–2019.

Sample ID	*gdh*	*bg*	*tpi*	Assigned Genotype
125	Negative	Negative	BIV	BIV
384	BIV	Negative	Negative	BIV
507	AII	AII	AII	AII
554	BIV	B	BIV	BIV
566	BIV	Negative	BIV	BIV
579	BIV	Negative	BIV	BIV
737	AII	AII	AII	AII
764	Negative	B	Negative	B
823	BIV	Negative	BIV	BIV
980	BIV	Negative	Negative	BIV
991	BIV	Negative	BIV	BIV
1030	BIV	Negative	Negative	BIV
1435	BIV	Negative	Negative	BIV
1459	BIV	B	Negative	BIV
1662	BIV	Negative	Negative	BIV
1721	BIV	B	BIV	BIV
1777	Negative	Negative	BIV	BIV
1784	BIV	B	Negative	BIV
1821	BIV	Negative	Negative	BIV
1904	BIV	Negative	Negative	BIV
1991	AII	AII	AII	AII
1997	AII	Negative	Negative	AII
2041	BIV	B	BIV	BIV
2172	BIII/BIV	Negative	Negative	BIII/BIV

**Table 3:** Diversity, frequency, and molecular features of *Giardia duodenalis* sequences at the *gdh*, *bg* and *tpi* loci obtained in the children population under study in Leganés, Madrid, 2017–2019. GenBank accession numbers are provided. Point mutations inducing amino acid substitutions are highlighted as superscript letters indicating the amino acid change.

Locus	Assemblage	Sub-Assemblage	Isolates	Reference Sequence	Stretch	Single Nucleotide Polymorphisms	GenBank ID
*gdh*	A	AII	3	L40510	64–496	None	MN844134
			1		76–491	T269Y^1^	MN844135
	B	BIV	6	L40508	76–491	None	MN844136
			1		76–496	C102Y, C105Y, C432Y, C435Y	MN844137
			1		78–485	C126Y	MN844138
			5		76–441	T183C, T387C, C396T, C423T	MN844139
			2		76–482	T183Y, T387Y, C396Y, C423Y	MN844140
			1		76–496	T183Y, T387Y, C423Y	MN844141
		BIII/BIV	1		76–496	C85Y^2^, T135Y, T183Y, G186R, C255Y, C273Y, C345Y, T366Y, C372Y, T387Y, A438R, G453R	MN844142
*bg*	A	AII	3	AY072723	96–604	None	MN844143
	B	B	3	AY072727	93–593	G159A, C165T, C309T, C324T, C393T, T471C	MN844144
			1		102–590	G159A, C165T, A265R^3^, C309T, C324T, C393T, T471C	MN844145
			1		93–604	G159A, C165T, C309T, C324T, C352T^4^, C393T, T471C	–
			1		102–604	G159A, C165T, C309T, C324T, C372T, C393T, T471C	MN844146
*tpi*	A	AII	2	U57897	282–751	None	MN844147
			1		276–805	C287G, A291W^5^	MN844148
	B	BIV	7	AF069560	1–479	None	MN844149
			1		1–479	A201R^6^	MN844150
			1		1–479	G305A, G425A, G426A^7^	MN844151

^1^ If C, pM90T; ^2^ If T, pP29S; ^3^ If G, pK89E; ^4^ Stop codon; ^5^ If T, pN95Y; ^6^ If G, pT74A; ^7^ pE143K; R: A/G; W: A/T; Y: C/T.

**Table 4:** Diversity, frequency, and molecular features of *Cryptosporidium* spp. sequences at the *ssu* rRNA locus obtained in the children population under study in Leganés, Madrid, 2017–2019. GenBank accession numbers are provided.

Species	No. of Isolates	Reference Sequence	Stretch	Single Nucleotide Polymorphisms	GenBank ID
*C. hominis*	5	AF108865	572–994	None	MN836820
	2		606–938	C607T, C620T	MN836821
	1		589–985	C607T, C620T, 697insT^1^, G862T	MN836822
	1		536–949	695_697delTTT^2^, T795C	MN836823
	1		687–952	A855G	MN836824
*C. parvum*	2	AF112571	603–877	Unknown^3^	–
	1		535–1025	A646G, T648C, T649G, 686-689delTAAT^2^, A691T, T910C	MN836825
*Cryptosporidium* spp.	1	–	–	–	–

^1^ ins: nucleotide insertion; ^2^ del: nucleotide deletion(s); ^3^ Sequences of insufficient quality to determine SNPs accurately.

## Supplementary Materials

Supplementary materials can be found at https://www.mdpi.com/2076-2607/8/4/466/s1.
