# Supplementary material for: Molecular Diversity of Giardia duodenalis, Cryptosporidium spp. and Blastocystis sp. in Asymptomatic School Children in Leganés, Madrid (Spain)
Source: Microorganisms. 2020 Mar 25;8(4):466. doi: 10.3390/microorganisms8040466 (PMC7232429; doi:10.3390/microorganisms8040466)
Supplement: Supplementary file 1 [file microorganisms-08-00466-s001.zip › microorganisms-728240-supplementary 8/Additional file 1 Table S1_Muadica_et_al.docx]

**Additional file 1: Table S1.** Oligonucleotides used for the molecular identification and/or characterization of *Giardia duodenalis*, *Cryptosporidium* spp., *Blastocystis* sp., and *Enterocytozoon bieneusi* in the present study.

| **Target organism** | **Locus** | **Oligonucleotide** | **Sequence (5´–3´)** | **Reference** |
| --- | --- | --- | --- | --- |
| *Giardia duodenalis* | *ssu* rRNA | Probe | FAM–CCCGCGGCGGTCCCTGCTAG–BHQ1 | [29] |
|  |  | Gd-80F | GACGGCTCAGGACAACGGTT | [29] |
|  |  | Gd-127R | TTGCCAGCGGTGTCCG | [29] |
|  | *gdh* | GDHeF | TCAACGTYAAYCGYGGYTTCCGT | [30] |
|  |  | GDHiF | CAGTACACCTCYGCTCTCGG | [30] |
|  |  | GDHiR | GTTRTCCTTGCACATCTCC | [30] |
|  | *bg* | G7_F | AAGCCCGACGACCTCACCCGCAGTGC | [31] |
|  |  | G759_R | GAGGCCGCCCTGGATCTTCGAGACGAC | [31] |
|  |  | G99_F | GAACGAACGAGATCGAGGTCCG | [31] |
|  |  | G609_R | CTCGACGAGCTTCGTGTT | [31] |
|  | *tpi* | AL3543_F | AAATIATGCCTGCTCGTCG | [32] |
|  |  | AL3546_R | CAAACCTTITCCGCAAACC | [32] |
|  |  | AL3544_F | CCCTTCATCGGIGGTAACTT | [32] |
|  |  | AL3545_R | GTGGCCACCACICCCGTGCC | [32] |
| *Cryptosporidium* spp. | *ssu* rRNA | CR-P1 | CAGGGAGGTAGTGACAAGAA | [33] |
|  |  | CR-P2 | TCAGCCTTGCGACCATACTC | [33] |
|  |  | CR-P3 | ATTGGAGGGCAAGTCTGGTG | [33] |
|  |  | CPB-DIAGR | TAAGGTGCTGAAGGAGTAAGG | [33] |
|  | *gp60* | AL-3531 | ATAGTCTCCGCTGTATTC | [34] |
|  |  | Al-3535 | GGAAGGAACGATGTATCT | [34] |
|  |  | AL-3532 | TCCGCTGTATTCTCAGCC | [34] |
|  |  | AL-3534 | GCAGAGGAACCAGCATC | [34] |
| *Blastocystis* sp. | *ssu* rRNA | BhRDr | GAGCTTTTTAACTGCAACAACG | [35] |
|  |  | RD5 | ATCTGGTTGATCCTGCCAGT | [35] |
| *Enterocytozoon bieneusi* | ITS | EBITS3_F | GGTCATAGGGATGAAGAG | [36] |
|  |  | EBITS4_R | TTCGAGTTCTTTCGCGCTC | [36] |
|  |  | EBITS1_F | GCTCTGAATATCTATGGCT | [36] |
|  |  | EBITS2.4_R | ATCGCCGACGGATCCAAGTG | [36] |
